# The Challenges Faced By Long-Term Care Residents and Their Families During COVID-19

**DOI:** 10.1093/geroni/igab046.1738

**Published:** 2021-12-17

**Authors:** Sharon Avidor, Liat Ayalon

**Affiliations:** 1 Ruppin Academic Center, Ruppin Academic Center, HaZafon, Israel; 2 Bar Ilan University, Ramat Gan, HaMerkaz, Israel

## Abstract

The present research aims to examine the effects of protective measures due to the coronavirus disease (COVID-19) within long-term care (LTC) settings on the residents and their family members. Open-ended qualitative interviews were conducted with 14 family members of older adults who resided in LTC settings during the first wave of the pandemic in Israel. The first theme identified is Rupture, including physical disconnect; the disruption in routine treatment to residents; and decline in the satisfaction with the setting. The second theme is Response, including sharing viewpoints and involvement in decision making, as well as an intense ambivalence shared by family members. Our findings highlight the distress caused to residents and family members by the isolation and restrictions in LTC settings during the pandemic, and underscore values and priorities that are central to them and their family members, including maintaining continuity, transparency, and working in unison with their families.

